# Terahertz Reconfigurable Planar Graphene Hybrid Yagi–Uda Antenna

**DOI:** 10.3390/nano15070488

**Published:** 2025-03-25

**Authors:** Qimeng Liu, Renbin Zhong, Boli Xu, Jiale Dong, Gefu Teng, Ke Zhong, Zhenhua Wu, Kaichun Zhang, Min Hu, Diwei Liu

**Affiliations:** Terahertz Research Center, School of Electronic Science and Engineering, Cooperative Innovation Centre of Terahertz Science, University of Electronic Science and Technology of China, Chengdu 611731, China; qmliu616@163.com (Q.L.); weibadiaole19990616@gmail.com (B.X.); dongjiale0530@163.com (J.D.); 202222021421@std.uestc.edu.cn (G.T.); jingxusihua@163.com (K.Z.); wuzhenhua@uestc.edu.cn (Z.W.); zhangkaichun@uestc.edu.cn (K.Z.); hu_m@uestc.edu.cn (M.H.); dwliu@uestc.edu.cn (D.L.)

**Keywords:** terahertz, reconfigurable, graphene, Yagi–Uda, antenna

## Abstract

In this paper, we design a frequency reconfigurable antenna for terahertz communication. The antenna is based on a Yagi design, with the main radiating elements being a pair of dipole antennas printed on the top and bottom of a dielectric substrate, respectively. The director and reflector elements give the antenna end-fire characteristics. The ends of the two arms of the dipole are constructed by staggered metal and graphene parasitic patches. By utilizing the effect of gate voltage on the conductivity of graphene, the equivalent length of the dipole antenna arms are altered and thereby adjust the antenna’s operating frequency. The proposed reconfigurable hybrid Yagi–Uda antenna can operate in five frequency bands separately at a peak gain of 4.53 dB. This reconfigurable antenna can meet the diverse requirements of the system without changing its structure and can reduce the size and cost while improving the performance.

## 1. Introduction

The terahertz (THz) band (frequency range of 0.1 to 10 THz) offers greater bandwidth and higher data rates compared to the RF and microwave bands [1]. A complete 137 GHz of bandwidth resources in terahertz bands were finalized and approved by the World Radiocommunication Conference 2019 (WRC-19) for both fixed and land mobile service applications [2].

Reconfigurable antennas can achieve a variety of performance characteristics without changing the antenna structure, enabling them to meet the diverse requirements of the system [3]. In the microwave and millimeter wave bands, PIN diodes are generally used to achieve antenna reconfigurability. However, they are unsuitable for the terahertz frequency band due to the low efficiency of rectifier diodes, mismatches between the diode and the antenna, and metal dispersion behavior [4,5,6,7].

Graphene-based THz reconfigurable antennas have been reported in recent years. In [8], a multifunctional terahertz dipole antenna is proposed that uses graphene and incorporates two tunable capacitive load loops as near-field resonant parasitic structures for multifunctional reconfigurability, but it has a gain of less than 2 dB, due to the considerable losses of graphene in the lower terahertz spectrum. This problem can be mitigated by using a hybrid structure of graphene and metal. In [9], a reconfigurable antenna combining metal and graphene is proposed, where metal serves as the main radiating element to improve radiation efficiency, and graphene parasitic patches are used to connect metal patches, providing tunability for the antenna. Compared to graphene antennas, their peak gain is 2.6 dB. The Yagi–Uda structure can increase the gain of an antenna [10]. The antenna proposed in this paper adopts the Yagi–Uda structure and has good performance. Moreover, it has the function of frequency reconfigurability and a peak gain of 4.53 dB.

This paper designed a frequency-reconfigurable Yagi antenna with metal–graphene structure. The antenna uses a pair of metal dipole antennas, printed on the top and bottom of a silica substrate, as the main radiators. The two arms of the dipole and the director are connected to multiple metal parasitic patches via graphene. By controlling the chemical potential of the graphene, it functions as a switch to adjust the equivalent lengths of the dipole antenna and the director, thereby changing the antenna’s operating frequencies.

## 2. Antenna Design

### 2.1. Graphene Modeling

Graphene is an extremely thin sheet that can be approximated as a two-dimensional material with equivalent surface electrical conductivity, denoted as *σ*. It depends on the operation frequency *ω*, the chemical potential *μ*, the relaxation time *τ*, and the temperature *T*. Generally, the electrical conductivity *σ* of graphene is primarily composed of intra-band conductivity $\sigma_{intra}$ and inter-band conductivity $\sigma_{inter}$, which can be described by the Kubo formula as follows:

(1) $$\sigma (\omega )=\sigma_{intra}(\omega )+\sigma_{inter}(\omega )$$

(2) $$\sigma_{intra}(\omega ,E_{F},\Gamma ,T)=\frac{-ie^{2}k_{B}T}{\pi \hbar^{2}(\omega -j2\Gamma )}\left[\frac{E_{F}}{k_{B}T}+2ln\left(exp(-\frac{E_{F}}{k_{B}T})+1\right)\right]$$

(3) $$\sigma_{inter}(\omega ,E_{F},\Gamma ,T)=\frac{-ie^{2}}{4\pi \hbar }\ln\left(\frac{2\left|E_{F}\right|-\left(\omega -i2\Gamma \right)\hbar }{2\left|E_{F}\right|+\left(\omega -i2\Gamma \right)\hbar }\right)$$

where *ω* is the angular frequency of the incident electromagnetic wave; *E*_*F*_ is the Fermi energy level; *e* is the charge of the electron; *Γ* = 0.5*τ* (*τ* is the electron relaxation time); *ħ* is the approximate Planck’s constant; *k*_*B*_ is Boltzmann’s constant; and *T* is the operation temperature. The Fermi energy level can be calculated as

(4) $$E_{F}\approx \hbar v_{F}\sqrt{\frac{\pi \varepsilon_{r}\varepsilon_{0}V_{g}}{eh_{d}}}$$

where *v*_*F*_ is the Fermi velocity, which is set as 106 m/s; *ε*_*r*_ is the relative dielectric constant of the medium; *ε*_0_ is the vacuum dielectric constant; ℎ_*d*_ is the substrate thickness. The Fermi energy level of graphene can be adjusted by bias voltage *V*_*g*_.

In the terahertz band, since the lower photon energy of terahertz waves is not capable of providing the energy required to excite the inter-band electron leaps (*E*_*F*_ ≫ *ħω*), the total electrical conductivity of graphene can be roughly determined by the graphene’s intra-band conductivity *σ*_*intra*_. So, it can be reduced to a characterization of the Drude model:

(5) $$\sigma_{intra}=\frac{e^{2}}{\pi \hbar^{2}}\frac{iE_{F}}{\left(\omega +i\tau^{-1}\right)}$$

With very small uniform thickness ∆, its planar equivalent dielectric constant is

(6) $$\varepsilon_{eff,t}=1+i\frac{\sigma_{s}}{\varepsilon_{0}\omega \Delta }$$

### 2.2. Frequency Reconfigurable Dipole Antenna Design

**Figure 1:**
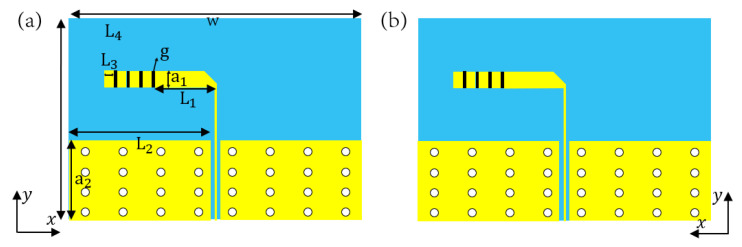
Schematic diagram of dipole antenna: (**a**) top view; (**b**) bottom view. The blue section corresponds to the silica dielectric substrate. The yellow section corresponds to metallic copper. The white section represents the vias.

**Figure 2:**
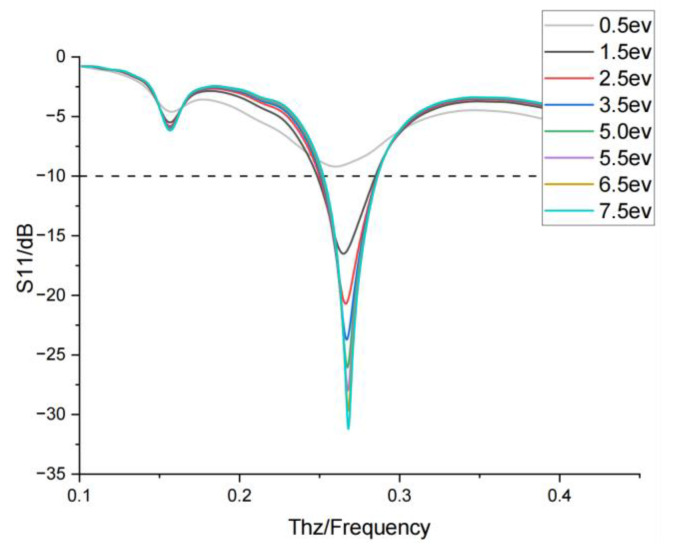
S11 parameters under different electrochemical potential values of graphene switches.

In this work, to achieve a tunable terahertz end-fire antenna with good performance, a dipole antenna loaded with graphene patches is first designed. The structure of the dipole antenna is shown in Figure 1 with one arm printed on the top of the SiO_2_ (Figure 1a) dielectric substrate and the other arm printed on the bottom (Figure 1b). The two arms of the dipole both have four discrete metal patches which can be connected via four graphene patches. They are numbered as g1, g2, g3, and g4 from the feeder line to the terminal. Controlling the switching of graphene patches can change the equivalent length of the dipole antenna, the electrochemical potential of graphene is set to 0 as the dielectric state, which is equivalent to “off”, and the electrochemical potential is set to 5 ev as the metallic state, which is equivalent to “on”, thus altering the resonant frequencies with five modes of M1, M2, M3, M4, and M5. To investigate the influence of graphene electrochemical potential variations on antenna performance, this study simulated the S11 parameter characteristics of an antenna under different graphene switch electrochemical potentials in the M1 operating mode. The results (as shown in Figure 2) demonstrate that when the graphene electrochemical potential approaches 0 eV, the antenna exhibits poor S11 performance, with graphene maintaining a dielectric state. As the electrochemical potential increases, the S11 characteristics significantly improve while the resonant frequency remains stable (Δf < 0.1 GHz) and the operational bandwidth shows minimal variations (within 5%). Based on this analysis, 5 eV is selected as the threshold electrochemical potential for activating the graphene switch.

The use of CPW (coplanar waveguide) feeds greatly reduces the reflection of electromagnetic waves. The current phase difference between the oscillator on the front and that on the back is 180°, acting as a broadband balun and broadening the bandwidth of the dipole antenna to some extent.

The main dimensional parameters of the dipole antenna are shown in Table 1. The bias voltage applied on different graphene patches for the five operation frequency modes are listed in Table 2, with mode 1 (M1) representing the longest arm and mode 5 (M5), the shortest arm. Obviously, the resonant frequencies of the dipole antenna will increase from M1 to M5 with the decreasing of the arm length.

**Figure 3:**
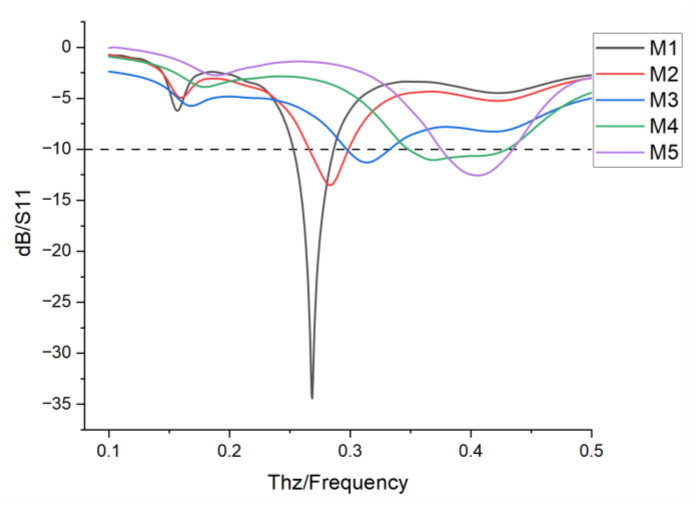
S11 curves of modes M1, M2, M3, M4, and M5.

The S11 curves in Figure 3 show that the −10 dB bandwidths of the dipole antenna for M1–M5 are 0.26–0.28 THz, 0.27–0.31 THz, 0.31–0.34 THz, 0.35–0.42 THz, and 0.37–0.44 THz, respectively, with a total span 0.18 THz. Its excellent performance makes it a suitable active driving oscillator for Yagi antennas.

### 2.3. Frequency Reconfigurable End-Fire Antenna Design

**Figure 4:**
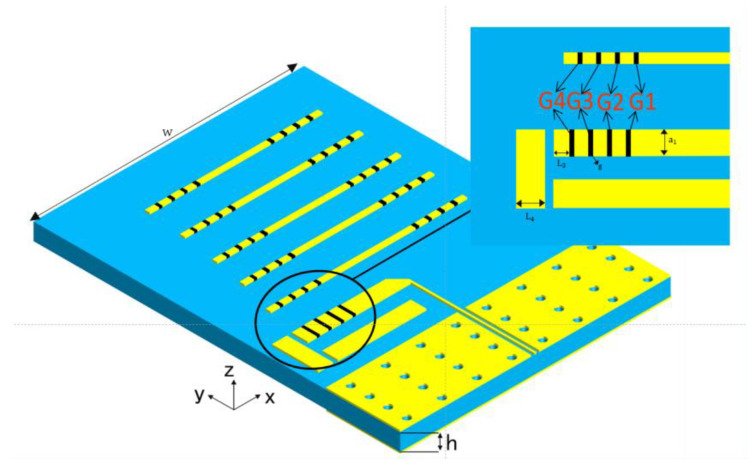
Three-dimensional antenna structure. The blue section corresponds to the silica dielectric substrate.

In order to enhance the electromagnetic wave coupling of the antenna, its two arms are patched with two parasitic copper strips at the end, respectively, as shown in Figure 4. Furthermore, the coplanar waveguide (CPW) section serves as a reflector and five parallel copper strips form the director. Then, a hybrid Yagi–Uda antenna is constructed, which is expected to achieve good gain and improved termination characteristics. To match the frequency-reconfigurable of the dipole antenna, the copper strips of the director are also equipped with four discrete copper patches symmetrically at both ends. The intervals of the patches are filled with graphene patches. The position arrangement and control of these patches are the same as the graphene patches in the radiation arms of the dipole antenna, as shown in Figure 4.

They are grouped as G1, G2, G3, and G4. The five operating modes with different graphene patches combination states are listed in Table 3, which indicates that the resonant frequency of the antenna progressively increases from 0.261 THz in M1 mode to 0.404 THz in M5 mode as both the equivalent arm length and the director length decrease.

**Figure 5:**
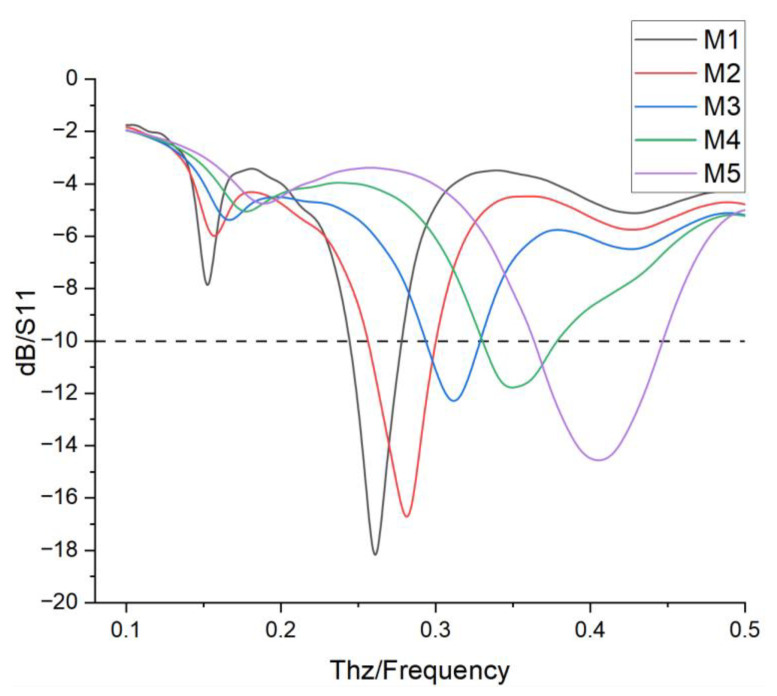
S11 curves for the five modes of the hybrid Yagi–Uda antenna.

The S11 parameters of the reconfigurable hybrid Yagi–Uda antenna are shown in Figure 5. For the five operation modes, the introduction of parasitic patches has broadened the antenna’s bandwidth. Specifically, when operating in the M1 mode, the resonant frequency is 0.261 THz with a −10 dB bandwidth of 0.243–0.278 THz. In the M2 mode, the resonant frequency is 0.281 THz with a −10 dB bandwidth of 0.256–0.299 THz. For the M3 mode, the resonant frequency is 0.311 THz and the −10 dB bandwidth is 0.296–0.325 THz. In the M4 mode, the resonant frequency is 0.351 THz with a −10 dB bandwidth of 0.329–0.378 THz. It can be observed that, for the M5 mode, the resonant frequency is 0.404 THz with a −10 dB bandwidth of 0.370–0.440 THz. However, for the M5 mode, it resonates at 0.404 THz and has the same bandwidth as that of the dipole antenna because the arm length at the M5 mode state is the shortest function just as that of the dipole antenna. Overall, the antenna’s −10 dB bandwidth spans from 0.243 to 0.440 THz, resulting in a relative bandwidth of 66.6%, which covers all the frequency bands allocated by WRC-19.

**Figure 6:**
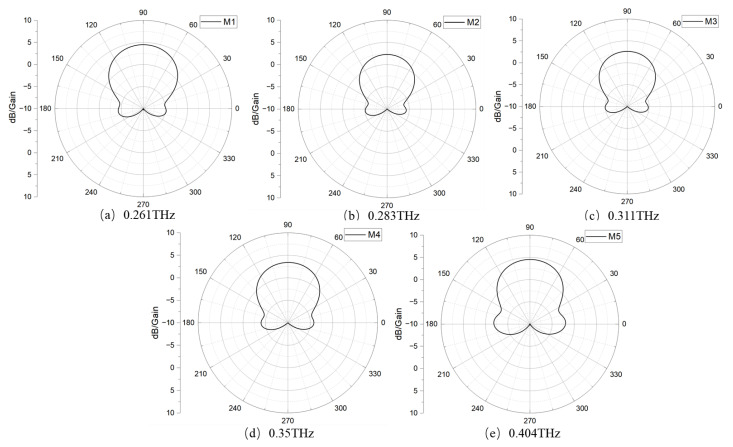
Gain diagram for the five modes.

**Figure 7:**
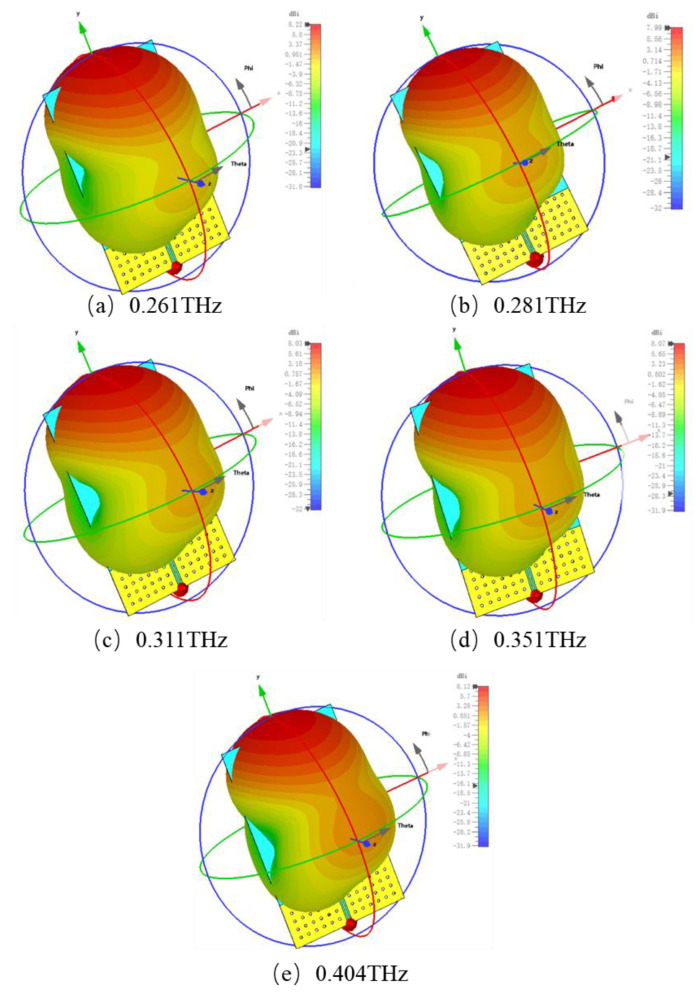
Three-dimensional orientation of the hybrid Yagi–Uda antenna for the five operating modes. The arrows represent the directionality coefficient of the maximum and minimum radiation directions.

The antenna gain for five operating modes are shown in Figure 6; they are 4.31 dB, 2.31 dB, 2.64 dB, 3.43 dB, and 4.53 dB, respectively, which exhibits improved gain and efficiency compared to the graphene Yagi antenna. Furthermore, the antenna demonstrates excellent end-fire performance as shown in Figure 7. The directivities for the five operating modes are 8.22 dBi, 7.99 dBi, 8.03 dBi, 8.07 dBi, and 8.12 dBi, respectively. Generally, the proposed hybrid Yagi–Uda antenna can offer an excellent tuning range from 0.243 to 0.444 THz with a gain exceeding 2.3 dB across the entire tuning range.

The proposed antenna is compared with the other literature in Table 4. The study by [11] proposes a graphene-based Yagi antenna with a good tuning range, but the high loss of graphene results in lower gain. The study by [12] presents a graphene antenna that achieves better gain, but the antenna may lack tuning capability. The study by [13] proposes a hybrid quasi-Yagi antenna, where the inclusion of a filter structure in the feed increases the complexity of the antenna. The study by [14] proposes a hybrid Yagi antenna with a small footprint and good gain. The proposed antenna in this paper demonstrates superior gain and efficiency compared to graphene-based radiating elements. While maintaining good gain and efficiency, it also exhibits an exceptional tunable range. This planar reconfigurable antenna features a structurally simple design with low fabrication costs and a highly feasible implementation. Characterized by excellent directivity, it presents significant potential for future applications in terahertz frequency band communications.

## 3. Conclusions

This work designs a frequency reconfigurable terahertz planar end-fire antenna based on a hybrid structure of graphene and metal. First, a frequency reconfigurable terahertz dipole antenna is proposed, which achieves a peak gain of 4.2 dBi at five frequencies. As an active driving element, a frequency-reconfigurable terahertz hybrid Yagi–Uda antenna is designed. It can operate in five frequency bands: 0.243–0.278 THz, 0.256–0.299 THz, 0.296–0.325 THz, 0.329–0.378 THz, and 0.244–0.444 THz. The relative bandwidth of the entire operating frequency range is 66.6% and its peak gain is 4.53 dBi, covering all frequency bands specified by WRC-19, indicating its great potential application for future terahertz communications.

## Figures and Tables

**Table 1:** Dimensional parameters of the antenna.

Parameters	Values	Parameters	Values
a1	30 μm	L2	333.5 μm
a2	70 μm	L3	20 μm
W	700 μm	L4	20 μm
L	500 μm	g	5 μm
L1	105 μm	a3	10 μm

**Table 2:** Five operation modes of dipole antennas.

Modes	g1	g2	g3	g4	Frequency
M1	5 ev	5 ev	5 ev	5 ev	0.268 THz
M2	0 ev	5 ev	5 ev	5 ev	0.283 THz
M3	0 ev	0 ev	5 ev	5 ev	0.311 THz
M4	0 ev	0 ev	0 ev	5 ev	0.395 THz
M5	0 ev	0 ev	0 ev	0 ev	0.416 THz

**Table 3:** Five operation modes of the hybrid Yagi–Uda antenna.

Modes	Group G1	Group G2	Group G3	Group G4	Frequency
M1	5 ev	5 ev	5 ev	5 ev	0.261 THz
M2	0 ev	5 ev	5 ev	5 ev	0.281 THz
M3	0 ev	0 ev	5 ev	5 ev	0.311 THz
M4	0 ev	0 ev	0 ev	5 ev	0.351 THz
M5	0 ev	0 ev	0 ev	0 ev	0.404 THz

**Table 4:** Five operation modes of antennas.

Reference	Antenna Structure	Size (λ × λ)	F (THz)	Peak Gain(dBi)	Tuning Range (THz)
[11]	Graphene	NR	0.95	1.13	0.7–0.98
[12]	Graphene	2.5 × 2.5	7.83	4.43	NR
[13]	PEC + Graphene	0.58 × 0.63	1.9	5	1.86–2.35
[14]	PEC + Graphene	0.44 × 0.27	1.36	4.93	1.328–1.5
This work	Copper + Graphene	0.58 × 1	0.261, 0.281, 0.311, 0.351, 0.404	4.53	0.243–0.444

## Data Availability

Data are available on request from the authors.

## References

[1] Kleine-Ostmann T., Nagatsuma T. (2011). A review on terahertz communications research. J. Infrared Millim. Terahertz Waves.

[2] Marcus M.J. (2019). ITU WRC-19 spectrum policy results. IEEE Wirel. Commun..

[3] Dash S., Patnaik A. (2018). Material selection for THz antennas. Microw. Opt. Technol. Lett..

[4] Kleiner R.J.S. (2007). Filling the terahertz gap. Science.

[5] Maurya N.K., Ghosh J. (2023). Design of graphene-based tunable ultra-thin UWB metasurface for terahertz regime. Optik.

[6] Maurya N.K., Kumari S., Pareek P., Singh L. (2023). Graphene-based frequency agile isolation enhancement mechanism for MIMO antenna in terahertz regime. Nano Commun. Netw..

[7] Nickpay M.-R., Danaie M., Shahzadi A. (2022). Wideband rectangular double-ring nanoribbon graphene-based antenna for terahertz communications. IETE J. Res..

[8] Fakharian M.M. (2022). A graphene-based multi-functional terahertz antenna. Optik.

[9] Moradi K., Pourziad A., Nikmehr S. (2021). A frequency reconfigurable microstrip antenna based on graphene in Terahertz Regime. Optik.

[10] Zarrabi F.B., Seyedsharbaty M.M., Ahmed Z., Arezoomand A.S., Heydari S. (2017). Wide band yagi antenna for terahertz application with graphene control. Optik.

[11] Chashmi M.J., Rezaei P., Kiani N. (2019). Reconfigurable graphene-based V-shaped dipole antenna: From quasi-isotropic to directional radiation pattern. Optik.

[12] Gotra S., Yadav R., Pandey V.S. (2020). Beam reconfigurable graphene-based Yagi–Uda antenna with higher-order TM mode generation for THz applications. Opt. Eng..

[13] Wu Y., Qu M., Jiao L., Liu Y. (2017). Tunable terahertz filter-integrated quasi-Yagi antenna based on graphene. Plasmonics.

[14] Maurya N.K., Kumari S., Pareek P., Varshney G. (2023). Highly-efficient tunable dipole-driven Yagi–Uda antenna with end-fire radiation for terahertz application. Nano Commun. Netw..

